# Ethyl 4,6-*O*-benzyl­idene-2-de­oxy-*N*-phthalimido-1-thio-β-d-glucopyran­oside

**DOI:** 10.1107/S1600536810047070

**Published:** 2010-11-20

**Authors:** Christoffer Hamark, Jens Landström, Lars Eriksson, Göran Widmalm

**Affiliations:** a Department of Organic Chemistry, Arrhenius Laboratory, Stockholm University, S-106 91 Stockholm, Sweden; b Department of Environmental and Material Chemistry, Arrhenius Laboratory, Stockholm University, S-106 91 Stockholm, Sweden

## Abstract

In the title compound, C_23_H_23_NO_6_S, the plane of the *N*-phthalimido group makes a dihedral angle of 67.4 (1)° with the least square plane of the sugar ring defined by the C2, C3, C5 and O5 atoms using standard glucose nomenclature. The thio­ethyl group has the *exo*-anomeric conformation. In the crystal, inter­molecular hydrogen bonds involving the hy­droxy groups and the carbonyl O atoms of adjacent *N*-phthalimido groups form chains parallel to the *b* axis. The chains are further stabilized by C—H⋯π inter­actions.

## Related literature

For the chemistry and applications of *N*-acetyl-β-d-glucosa­mine derivatives, see: Tan *et al.* (2009 ▶); Werz *et al.* (2007 ▶). For the conformation of related compounds, see: Lemieux & Koto (1974 ▶); Färnbäck *et al.* (2007 ▶). For the synthesis of the title compound, see: Lönn (1985 ▶). For puckering parameters, see: Cremer & Pople (1975 ▶). 
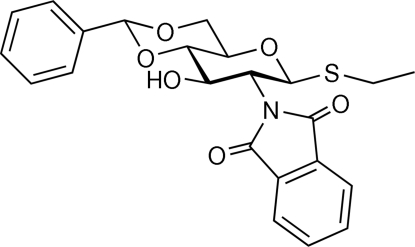


## Experimental

### 

#### Crystal data

C_23_H_23_NO_6_S
*M* *_r_* = 441.48
Orthorhombic, 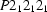

*a* = 8.6728 (6) Å
*b* = 9.7583 (10) Å
*c* = 25.3102 (15) Å
*V* = 2142.0 (3) Å^3^
*Z* = 4
Mo *K*α radiation
μ = 0.19 mm^−1^
*T* = 293 K
0.30 × 0.12 × 0.05 mm

#### Data collection

Stoe IPDS diffractometer
Absorption correction: numerical (*X-RED*; Stoe & Cie, 1997) ▶ *T*_min_ = 0.730, *T*_max_ = 0.933
12985 measured reflections
5120 independent reflections
2352 reflections with *I* > 2σ(*I*)
*R*_int_ = 0.110

#### Refinement

*R*[*F* ^2^ > 2σ(*F* ^2^)] = 0.036
*wR*(*F* ^2^) = 0.110
*S* = 0.83
5120 reflections
281 parameters
H-atom parameters constrained
Δρ_max_ = 0.21 e Å^−3^
Δρ_min_ = −0.27 e Å^−3^
Absolute structure: Flack (1983 ▶), 1544 Friedel pairs
Flack parameter: −0.07 (10)

### 

Data collection: *EXPOSE* (Stoe & Cie, 1997) ▶; cell refinement: *CELL* (Stoe & Cie, 1997) ▶; data reduction: *INTEGRATE* (Stoe & Cie, 1997) ▶; program(s) used to solve structure: *SHELXS97* (Sheldrick, 2008 ▶); program(s) used to refine structure: *SHELXL97* (Sheldrick, 2008 ▶); molecular graphics: *DIAMOND* (Brandenburg, 1999 ▶); software used to prepare material for publication: *PLATON* (Spek, 2009 ▶).

## Supplementary Material

Crystal structure: contains datablocks global, I. DOI: 10.1107/S1600536810047070/rz2504sup1.cif

Structure factors: contains datablocks I. DOI: 10.1107/S1600536810047070/rz2504Isup2.hkl

Additional supplementary materials:  crystallographic information; 3D view; checkCIF report

## Figures and Tables

**Table 1:** Hydrogen-bond geometry (Å, °) *C*g is the centroid of the C23—C28 ring.

*D*—H⋯*A*	*D*—H	H⋯*A*	*D*⋯*A*	*D*—H⋯*A*
O3—H3*A*⋯O30^i^	0.82	2.27	3.014 (3)	150
C14—H14⋯*Cg*^i^	0.93	2.98	3.613 (3)	126

Symmetry code: (i) 
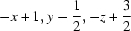
.

**Fig. 1.**
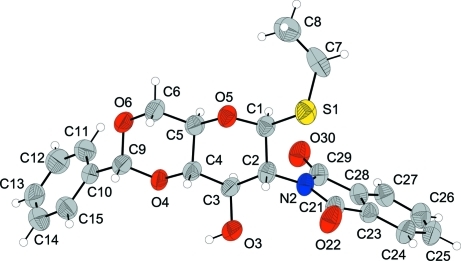
Molecular structure showing 50% probability displacement ellipsoids.

**Fig. 2.**
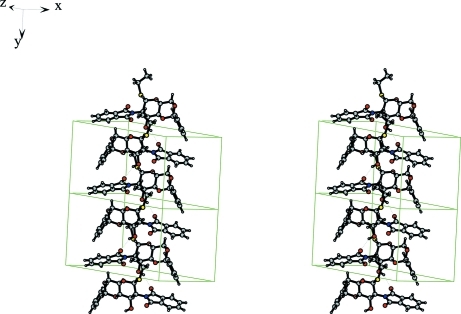
Stereoview showing the intermolecular hydrogen bond intercations between molecules forming a chain along the b-direction.

## supplementary crystallographic information

### Comment

*N*-acetyl-D-glucosamine (D-GlcNAc) is found in nature in bacteria, crustaceans as well as in mammals. In glycoproteins the β-D-GlcNAc is present in N-linked oligosaccharides and it is of great importance to have access to a large arsenal of different suitably protected synthetic precursors in order to carry out synthesis of a variety of different oligosaccharides (Werz *et al.* 2007). These can be used as probes in microarray applications or to synthesize N-linked glycoproteins (Tan *et al.* 2009). In the structure shown in Fig. 1 the least square plane of the N-phthaloyl group makes a dihedral angle of 67.4 (1)° to the sugar ring plane defined by the four atoms (C2,C3,C5,O5).

In glycosides the φ torsion angle (H1—C1—S1—C7) is of particular interest and is for the title compound in agreement with the *exo*-anomeric effect (Lemieux & Koto, 1974). It is, however sligthly shifted away from a staggered conformation, to 25.4°. The Cremer & Pople (1975) parameters for the different rings are for (O5—C5): Q=0.585 (3) Å, θ=8.4 (3)° and φ=329 (2)°, for (O4,C4,C5,C6,O6,C9): Q=0.575 (3) Å, θ=3.4 (3)° and φ=126 (4)°. These Q-values are similar to total puckering amplitudes for previously described pyranosides (Färnbäck *et al.*, 2007).

Intermolecular hydrogen bonding from the hydroxy group is present (Table 1) where one of the carbonyl O atoms in the N-phthaloyl group act as acceptor, making up chains along the [010] direction shown in Fig. 2. In addition to this conventional hydrogen bond the intermolecular packing is stabilized due to interactions between substituents of the sugar rings. There is a salient C—H···π interaction between the center of gravity (*Cg*) of the ring C23—C28 of the N-phthaloyl group and the *meta* position (C14) of the phenyl group (C10—C15). Furthermore there are three more π···π interactions present with d(*Cg*—*Cg*) < 4.8 Å with dihedral angles between the interacting π systems in the vicinity of 60° indicating a herringbone packing pattern.

### Experimental

The synthesis of the title compound has been described previously (Lönn, 1985). Colourless crystals of the title compound were grown from diethyl ether/pentane (1:1 *v*/*v*) at ambient temperature.

### Refinement

The hydrogen atoms were refined in riding mode with C–H = 0.93–0.98 Å, O–H = 0.82 Å, and with *U*_iso_(H) = 1.2*U*_eq_(C) or 1.5*U*_eq_(C, O) for methyl and hydroxy H atoms.

### Crystal data

**Table:**

C_23_H_23_NO_6_S	*F*(000) = 928
*M**_r_* = 441.48	*D*_x_ = 1.369 Mg m^−^^3^
Orthorhombic, *P*2_1_2_1_2_1_	Mo *K*α radiation, λ = 0.71073 Å
Hall symbol: P 2ac 2ab	Cell parameters from 5000 reflections
*a* = 8.6728 (6) Å	θ = 2.4–23.3°
*b* = 9.7583 (10) Å	µ = 0.19 mm^−^^1^
*c* = 25.3102 (15) Å	*T* = 293 K
*V* = 2142.0 (3) Å^3^	Prism, colourless
*Z* = 4	0.30 × 0.12 × 0.05 mm

### Data collection

**Table:**

Stoe IPDS diffractometer	3734 independent reflections
Radiation source: fine-focus sealed tube	2352 reflections with *I* > 2σ(*I*)
graphite	*R*_int_ = 0.110
Detector resolution: 6.7 pixels mm^-1^	θ_max_ = 25.0°, θ_min_ = 2.2°
φ scan	*h* = −10→10
Absorption correction: numerical (*X-RED*; Stoe & Cie, 1997)	*k* = −11→11
*T*_min_ = 0.730, *T*_max_ = 0.933	*l* = −29→29
5120 measured reflections	

### Refinement

**Table:**

Refinement on *F*^2^	Secondary atom site location: difference Fourier map
Least-squares matrix: full	Hydrogen site location: inferred from neighbouring sites
*R*[*F*^2^ > 2σ(*F*^2^)] = 0.036	H-atom parameters constrained
*wR*(*F*^2^) = 0.110	*w* = 1/[σ^2^(*F*_o_^2^) + (0.0371*P*)^2^] where *P* = (*F*_o_^2^ + 2*F*_c_^2^)/3
*S* = 0.83	(Δ/σ)_max_ < 0.001
5120 reflections	Δρ_max_ = 0.21 e Å^−^^3^
281 parameters	Δρ_min_ = −0.27 e Å^−^^3^
0 restraints	Absolute structure: Flack (1983), 1544 Friedel pairs
Primary atom site location: structure-invariant direct methods	Flack parameter: −0.07 (10)

### Special details

**Table:**

Geometry. All e.s.d.'s (except the e.s.d. in the dihedral angle between two l.s. planes) are estimated using the full covariance matrix. The cell e.s.d.'s are taken into account individually in the estimation of e.s.d.'s in distances, angles and torsion angles; correlations between e.s.d.'s in cell parameters are only used when they are defined by crystal symmetry. An approximate (isotropic) treatment of cell e.s.d.'s is used for estimating e.s.d.'s involving l.s. planes.
Refinement. Refinement of *F*^2^ against ALL reflections. The weighted *R*-factor *wR* and goodness of fit *S* are based on *F*^2^, conventional *R*-factors *R* are based on *F*, with *F* set to zero for negative *F*^2^. The threshold expression of *F*^2^ > σ(*F*^2^) is used only for calculating *R*-factors(gt) *etc*. and is not relevant to the choice of reflections for refinement. *R*-factors based on *F*^2^ are statistically about twice as large as those based on *F*, and *R*- factors based on ALL data will be even larger.

### Fractional atomic coordinates and isotropic or equivalent isotropic displacement parameters (Å^2^)

**Table:**

	*x*	*y*	*z*	*U*_iso_*/*U*_eq_	
S1	0.32966 (10)	0.49384 (9)	0.89236 (3)	0.0598 (2)	
C7	0.4033 (4)	0.6664 (4)	0.88601 (16)	0.0742 (11)	
H7A	0.4218	0.7027	0.9211	0.089*	
H7B	0.5018	0.6626	0.8679	0.089*	
C8	0.2991 (6)	0.7648 (4)	0.85654 (18)	0.0869 (13)	
H8A	0.3469	0.8535	0.8550	0.130*	
H8B	0.2021	0.7719	0.8746	0.130*	
H8C	0.2822	0.7315	0.8213	0.130*	
C1	0.3502 (4)	0.4316 (3)	0.82624 (11)	0.0449 (7)	
H1	0.4327	0.4826	0.8085	0.054*	
C2	0.3910 (3)	0.2767 (3)	0.82645 (11)	0.0438 (7)	
H2	0.3098	0.2297	0.8464	0.053*	
C3	0.3922 (3)	0.2146 (3)	0.77071 (11)	0.0440 (7)	
H3	0.4823	0.2486	0.7513	0.053*	
C4	0.2480 (3)	0.2552 (3)	0.74190 (11)	0.0425 (7)	
H4	0.1591	0.2098	0.7582	0.051*	
C5	0.2252 (4)	0.4098 (3)	0.74389 (12)	0.0475 (8)	
H5	0.3148	0.4557	0.7283	0.057*	
O5	0.2082 (2)	0.45149 (19)	0.79800 (8)	0.0495 (5)	
O3	0.4053 (3)	0.0694 (2)	0.77736 (10)	0.0681 (7)	
H3A	0.3456	0.0306	0.7572	0.102*	
O4	0.2607 (2)	0.21320 (19)	0.68835 (8)	0.0463 (5)	
C9	0.1286 (4)	0.2511 (3)	0.65856 (12)	0.0504 (8)	
H9	0.0378	0.2032	0.6723	0.061*	
O6	0.1033 (3)	0.39409 (19)	0.66026 (8)	0.0576 (6)	
C6	0.0822 (4)	0.4450 (3)	0.71277 (13)	0.0558 (8)	
H6A	−0.0077	0.4030	0.7288	0.067*	
H6B	0.0670	0.5435	0.7121	0.067*	
C10	0.1571 (4)	0.2085 (3)	0.60276 (12)	0.0470 (7)	
C11	0.2464 (4)	0.2843 (3)	0.56915 (14)	0.0632 (10)	
H11	0.2842	0.3687	0.5804	0.076*	
C12	0.2818 (5)	0.2385 (4)	0.51886 (15)	0.0731 (10)	
H12	0.3437	0.2910	0.4967	0.088*	
C13	0.2247 (5)	0.1146 (3)	0.50192 (15)	0.0688 (10)	
H13	0.2474	0.0830	0.4681	0.083*	
C14	0.1343 (5)	0.0382 (3)	0.53503 (15)	0.0699 (10)	
H14	0.0957	−0.0458	0.5237	0.084*	
C15	0.1001 (4)	0.0850 (3)	0.58515 (14)	0.0594 (9)	
H15	0.0379	0.0326	0.6072	0.071*	
N2	0.5361 (3)	0.2509 (2)	0.85380 (9)	0.0438 (6)	
C21	0.5417 (4)	0.1857 (3)	0.90323 (12)	0.0455 (7)	
O22	0.4289 (3)	0.1431 (2)	0.92638 (9)	0.0658 (7)	
C23	0.7059 (4)	0.1797 (3)	0.91881 (12)	0.0447 (7)	
C24	0.7748 (4)	0.1282 (3)	0.96358 (13)	0.0545 (8)	
H24	0.7167	0.0916	0.9911	0.065*	
C25	0.9354 (4)	0.1333 (3)	0.96599 (14)	0.0589 (9)	
H25	0.9859	0.0994	0.9957	0.071*	
C26	1.0203 (4)	0.1878 (4)	0.92502 (15)	0.0598 (9)	
H26	1.1273	0.1871	0.9271	0.072*	
C27	0.9503 (4)	0.2434 (3)	0.88077 (12)	0.0528 (8)	
H27	1.0077	0.2830	0.8537	0.063*	
C28	0.7922 (3)	0.2375 (3)	0.87864 (11)	0.0446 (7)	
C29	0.6829 (4)	0.2885 (3)	0.83770 (12)	0.0468 (7)	
O30	0.7131 (3)	0.3513 (2)	0.79655 (9)	0.0606 (6)	

### Atomic displacement parameters (Å^2^)

**Table:**

	*U*^11^	*U*^22^	*U*^33^	*U*^12^	*U*^13^	*U*^23^
S1	0.0730 (6)	0.0708 (5)	0.0354 (5)	0.0043 (4)	0.0044 (4)	−0.0071 (4)
C7	0.063 (2)	0.092 (3)	0.068 (3)	−0.021 (2)	0.005 (2)	−0.033 (2)
C8	0.114 (4)	0.069 (2)	0.078 (3)	−0.014 (2)	−0.005 (3)	0.000 (2)
C1	0.0474 (19)	0.0512 (16)	0.0360 (18)	0.0038 (13)	0.0010 (14)	−0.0006 (13)
C2	0.0416 (17)	0.0561 (17)	0.0338 (18)	0.0013 (13)	0.0009 (13)	0.0043 (13)
C3	0.0504 (18)	0.0451 (16)	0.0364 (18)	0.0125 (13)	−0.0011 (13)	0.0021 (12)
C4	0.0486 (17)	0.0449 (15)	0.0340 (18)	0.0057 (13)	0.0002 (12)	0.0043 (13)
C5	0.060 (2)	0.0466 (16)	0.0355 (19)	0.0066 (14)	0.0006 (15)	−0.0020 (12)
O5	0.0534 (13)	0.0574 (12)	0.0377 (13)	0.0118 (9)	−0.0016 (10)	−0.0038 (9)
O3	0.0912 (19)	0.0507 (12)	0.0624 (18)	0.0218 (12)	−0.0265 (13)	−0.0069 (10)
O4	0.0524 (12)	0.0512 (11)	0.0352 (13)	0.0086 (9)	−0.0091 (9)	−0.0031 (8)
C9	0.057 (2)	0.0474 (17)	0.047 (2)	0.0087 (15)	−0.0077 (15)	0.0031 (13)
O6	0.0801 (16)	0.0517 (13)	0.0410 (14)	0.0189 (11)	−0.0137 (11)	−0.0019 (9)
C6	0.063 (2)	0.0601 (19)	0.044 (2)	0.0174 (16)	−0.0084 (16)	0.0004 (14)
C10	0.0546 (18)	0.0479 (16)	0.0385 (19)	0.0018 (14)	−0.0114 (15)	0.0027 (13)
C11	0.089 (3)	0.0551 (19)	0.046 (2)	−0.0067 (18)	−0.0045 (18)	0.0008 (15)
C12	0.096 (3)	0.073 (2)	0.050 (3)	−0.008 (2)	0.001 (2)	0.0095 (18)
C13	0.095 (3)	0.068 (2)	0.043 (2)	0.011 (2)	−0.013 (2)	−0.0061 (17)
C14	0.096 (3)	0.060 (2)	0.054 (3)	−0.0034 (19)	−0.014 (2)	−0.0097 (17)
C15	0.066 (2)	0.0569 (19)	0.055 (2)	−0.0103 (17)	−0.0049 (18)	0.0005 (15)
N2	0.0399 (14)	0.0605 (15)	0.0311 (15)	−0.0018 (12)	−0.0001 (11)	0.0039 (11)
C21	0.0474 (19)	0.0567 (17)	0.0324 (19)	−0.0021 (14)	0.0028 (14)	0.0055 (12)
O22	0.0535 (14)	0.0927 (17)	0.0513 (15)	−0.0082 (12)	0.0001 (12)	0.0279 (12)
C23	0.0504 (19)	0.0476 (15)	0.0361 (18)	0.0017 (14)	−0.0011 (14)	0.0023 (13)
C24	0.059 (2)	0.066 (2)	0.038 (2)	0.0025 (16)	−0.0042 (16)	0.0088 (15)
C25	0.058 (2)	0.072 (2)	0.047 (2)	0.0102 (17)	−0.0126 (18)	0.0021 (16)
C26	0.0464 (19)	0.074 (2)	0.059 (2)	0.0033 (17)	−0.0078 (17)	−0.0069 (18)
C27	0.055 (2)	0.0589 (19)	0.045 (2)	−0.0040 (16)	−0.0001 (15)	−0.0058 (14)
C28	0.0436 (17)	0.0522 (16)	0.0379 (19)	−0.0018 (14)	0.0003 (13)	−0.0043 (13)
C29	0.0509 (19)	0.0556 (18)	0.0339 (19)	−0.0004 (14)	0.0025 (14)	−0.0011 (13)
O30	0.0596 (14)	0.0801 (15)	0.0423 (14)	−0.0080 (11)	0.0040 (11)	0.0159 (11)

### Geometric parameters (Å, °)

**Table:**

S1—C1	1.789 (3)	C6—H6A	0.9700
S1—C7	1.808 (4)	C6—H6B	0.9700
C7—C8	1.515 (6)	C10—C11	1.368 (5)
C7—H7A	0.9700	C10—C15	1.376 (4)
C7—H7B	0.9700	C11—C12	1.384 (5)
C8—H8A	0.9600	C11—H11	0.9300
C8—H8B	0.9600	C12—C13	1.374 (5)
C8—H8C	0.9600	C12—H12	0.9300
C1—O5	1.437 (4)	C13—C14	1.369 (5)
C1—C2	1.553 (4)	C13—H13	0.9300
C1—H1	0.9800	C14—C15	1.381 (5)
C2—N2	1.458 (4)	C14—H14	0.9300
C2—C3	1.535 (4)	C15—H15	0.9300
C2—H2	0.9800	N2—C29	1.387 (4)
C3—O3	1.431 (3)	N2—C21	1.404 (4)
C3—C4	1.501 (4)	C21—O22	1.214 (4)
C3—H3	0.9800	C21—C23	1.479 (4)
C4—O4	1.420 (3)	C23—C24	1.376 (4)
C4—C5	1.523 (4)	C23—C28	1.383 (4)
C4—H4	0.9800	C24—C25	1.395 (5)
C5—O5	1.436 (4)	C24—H24	0.9300
C5—C6	1.509 (4)	C25—C26	1.378 (5)
C5—H5	0.9800	C25—H25	0.9300
O3—H3A	0.8200	C26—C27	1.385 (5)
O4—C9	1.420 (4)	C26—H26	0.9300
C9—O6	1.413 (3)	C27—C28	1.373 (5)
C9—C10	1.493 (4)	C27—H27	0.9300
C9—H9	0.9800	C28—C29	1.490 (4)
O6—C6	1.430 (4)	C29—O30	1.236 (4)
			
C1—S1—C7	101.41 (16)	C9—O6—C6	113.0 (2)
C8—C7—S1	115.0 (3)	O6—C6—C5	107.5 (3)
C8—C7—H7A	108.5	O6—C6—H6A	110.2
S1—C7—H7A	108.5	C5—C6—H6A	110.2
C8—C7—H7B	108.5	O6—C6—H6B	110.2
S1—C7—H7B	108.5	C5—C6—H6B	110.2
H7A—C7—H7B	107.5	H6A—C6—H6B	108.5
C7—C8—H8A	109.5	C11—C10—C15	118.4 (3)
C7—C8—H8B	109.5	C11—C10—C9	122.1 (3)
H8A—C8—H8B	109.5	C15—C10—C9	119.4 (3)
C7—C8—H8C	109.5	C10—C11—C12	121.5 (3)
H8A—C8—H8C	109.5	C10—C11—H11	119.3
H8B—C8—H8C	109.5	C12—C11—H11	119.3
O5—C1—C2	109.2 (2)	C13—C12—C11	119.4 (4)
O5—C1—S1	109.53 (19)	C13—C12—H12	120.3
C2—C1—S1	110.5 (2)	C11—C12—H12	120.3
O5—C1—H1	109.2	C14—C13—C12	119.6 (4)
C2—C1—H1	109.2	C14—C13—H13	120.2
S1—C1—H1	109.2	C12—C13—H13	120.2
N2—C2—C3	111.2 (2)	C13—C14—C15	120.3 (3)
N2—C2—C1	111.5 (2)	C13—C14—H14	119.8
C3—C2—C1	112.5 (2)	C15—C14—H14	119.8
N2—C2—H2	107.1	C10—C15—C14	120.7 (3)
C3—C2—H2	107.1	C10—C15—H15	119.7
C1—C2—H2	107.1	C14—C15—H15	119.7
O3—C3—C4	112.6 (2)	C29—N2—C21	110.5 (2)
O3—C3—C2	106.5 (2)	C29—N2—C2	127.4 (2)
C4—C3—C2	109.6 (2)	C21—N2—C2	122.1 (2)
O3—C3—H3	109.4	O22—C21—N2	123.9 (3)
C4—C3—H3	109.4	O22—C21—C23	129.3 (3)
C2—C3—H3	109.4	N2—C21—C23	106.8 (2)
O4—C4—C3	108.8 (2)	C24—C23—C28	121.3 (3)
O4—C4—C5	109.1 (2)	C24—C23—C21	130.7 (3)
C3—C4—C5	110.7 (2)	C28—C23—C21	108.0 (3)
O4—C4—H4	109.4	C23—C24—C25	117.2 (3)
C3—C4—H4	109.4	C23—C24—H24	121.4
C5—C4—H4	109.4	C25—C24—H24	121.4
O5—C5—C6	110.4 (3)	C26—C25—C24	120.9 (3)
O5—C5—C4	109.0 (2)	C26—C25—H25	119.5
C6—C5—C4	108.3 (3)	C24—C25—H25	119.5
O5—C5—H5	109.7	C25—C26—C27	121.7 (3)
C6—C5—H5	109.7	C25—C26—H26	119.1
C4—C5—H5	109.7	C27—C26—H26	119.1
C5—O5—C1	110.4 (2)	C28—C27—C26	116.9 (3)
C3—O3—H3A	109.5	C28—C27—H27	121.5
C4—O4—C9	111.6 (2)	C26—C27—H27	121.5
O6—C9—O4	111.5 (2)	C27—C28—C23	121.9 (3)
O6—C9—C10	109.3 (2)	C27—C28—C29	130.4 (3)
O4—C9—C10	107.2 (2)	C23—C28—C29	107.6 (3)
O6—C9—H9	109.6	O30—C29—N2	125.0 (3)
O4—C9—H9	109.6	O30—C29—C28	128.1 (3)
C10—C9—H9	109.6	N2—C29—C28	106.9 (2)
			
C7—S1—C1—H1	25.4	C9—C10—C11—C12	174.9 (3)
C1—S1—C7—C8	72.3 (3)	C10—C11—C12—C13	0.7 (6)
C7—S1—C1—O5	−94.1 (2)	C11—C12—C13—C14	−0.3 (6)
C7—S1—C1—C2	145.6 (2)	C12—C13—C14—C15	0.2 (6)
O5—C1—C2—N2	178.9 (2)	C11—C10—C15—C14	0.9 (5)
S1—C1—C2—N2	−60.6 (3)	C9—C10—C15—C14	−175.1 (3)
O5—C1—C2—C3	53.1 (3)	C13—C14—C15—C10	−0.5 (6)
S1—C1—C2—C3	173.7 (2)	C3—C2—N2—C29	58.1 (4)
N2—C2—C3—O3	63.6 (3)	C1—C2—N2—C29	−68.4 (4)
C1—C2—C3—O3	−170.5 (2)	C3—C2—N2—C21	−124.5 (3)
N2—C2—C3—C4	−174.3 (2)	C1—C2—N2—C21	109.0 (3)
C1—C2—C3—C4	−48.4 (3)	C29—N2—C21—O22	179.7 (3)
O3—C3—C4—O4	−69.7 (3)	C2—N2—C21—O22	1.9 (5)
C2—C3—C4—O4	172.0 (2)	C29—N2—C21—C23	−1.2 (3)
O3—C3—C4—C5	170.4 (2)	C2—N2—C21—C23	−179.0 (2)
C2—C3—C4—C5	52.1 (3)	O22—C21—C23—C24	−1.8 (6)
O4—C4—C5—O5	178.5 (2)	N2—C21—C23—C24	179.1 (3)
C3—C4—C5—O5	−61.8 (3)	O22—C21—C23—C28	177.8 (3)
O4—C4—C5—C6	58.2 (3)	N2—C21—C23—C28	−1.2 (3)
C3—C4—C5—C6	178.0 (3)	C28—C23—C24—C25	−1.8 (5)
C6—C5—O5—C1	−173.8 (2)	C21—C23—C24—C25	177.8 (3)
C4—C5—O5—C1	67.3 (3)	C23—C24—C25—C26	0.0 (5)
C2—C1—O5—C5	−62.4 (3)	C24—C25—C26—C27	2.1 (5)
S1—C1—O5—C5	176.51 (19)	C25—C26—C27—C28	−2.3 (5)
C3—C4—O4—C9	−178.8 (2)	C26—C27—C28—C23	0.4 (5)
C5—C4—O4—C9	−57.9 (3)	C26—C27—C28—C29	179.1 (3)
C4—O4—C9—O6	57.8 (3)	C24—C23—C28—C27	1.6 (5)
C4—O4—C9—C10	177.3 (2)	C21—C23—C28—C27	−178.1 (3)
O4—C9—O6—C6	−58.9 (3)	C24—C23—C28—C29	−177.3 (3)
C10—C9—O6—C6	−177.2 (3)	C21—C23—C28—C29	3.0 (3)
C9—O6—C6—C5	58.9 (3)	C21—N2—C29—O30	−177.7 (3)
O5—C5—C6—O6	−176.8 (2)	C2—N2—C29—O30	0.0 (5)
C4—C5—C6—O6	−57.5 (3)	C21—N2—C29—C28	3.0 (3)
O6—C9—C10—C11	41.1 (4)	C2—N2—C29—C28	−179.4 (3)
O4—C9—C10—C11	−79.8 (3)	C27—C28—C29—O30	−1.9 (5)
O6—C9—C10—C15	−143.0 (3)	C23—C28—C29—O30	177.0 (3)
O4—C9—C10—C15	96.1 (3)	C27—C28—C29—N2	177.4 (3)
C15—C10—C11—C12	−1.1 (5)	C23—C28—C29—N2	−3.7 (3)

### Hydrogen-bond geometry (Å, °)

**Table:**

*C*g is the centroid of the C23—C28 ring.

**Table:**

*D*—H···*A*	*D*—H	H···*A*	*D*···*A*	*D*—H···*A*
O3—H3A···O30^i^	0.82	2.27	3.014 (3)	150
C14—H14···Cg^i^	0.93	2.98	3.613 (3)	126

Symmetry codes: (i) −*x*+1, *y*−1/2, −*z*+3/2.
